# Tokophobia and associated factors in pregnancy: A community-based cross-sectional survey

**DOI:** 10.1371/journal.pone.0345900

**Published:** 2026-06-02

**Authors:** Adeniyi Abiodun Adewunmi, Lateef Adekunle Akinola, Ayokunle Moses Olumodeji, Gideon Ntieno Inyangudo, Tawaqualit Abimbola Ottun, Abiodun Samuel Adegoke, Justina Ucheojor Onwuka, Bolanle Adeyemi Ola

**Affiliations:** 1 Department of Obstetrics and Gynaecology, Lagos State University College of Medicine, Lagos, Nigeria; 2 Department of Obstetrics and Gynaecology, Lagos State University Teaching Hospital, Lagos, Nigeria; 3 Medison Specialist Women Hospital, Lagos, Nigeria; 4 School of Public Health, University of Port Harcourt, Port Harcourt, Nigeria; 5 Department of Behavioural Medicine, Lagos State University College of Medicine, Lagos, Nigeria; Federal Medical Centre Birnin Kudu, NIGERIA

## Abstract

**Background:**

Fear of childbirth (tokophobia) impacts maternal health, yet its prevalence and associated factors remain underexplored in Lagos. This study aimed to determine the prevalence of tokophobia and its associated risk and psychological factors.

**Methods:**

This community-based cross-sectional survey, conducted in Lagos, over a 4-month period, involved 855 pregnant women attending antenatal care at Lagos’ 57 flagship primary health care centers (PHCs). Fifteen eligible pregnant women who were consecutively selected from each PHC’s antenatal clinic list, had in-depth interviews using a structured questionnaire consisting of the Wijma Delivery Expectancy Questionnaire (W-DEQ A) for fear of childbirth, the Depression Anxiety Stress Scales (DASS-21), and the Generalized Anxiety Disorder 7-item (GAD-7) scale. Data was analyzed with appropriate descriptive and inferential statistics including trend-based tests for ordinal variables and multivariable logistic regression modeling ordinal predictors at <0.05 significance level.

**Results:**

The women’s mean age was 28.37 years and majority were multigravida, in their third trimester with secondary level of education. Fear of childbirth levels revealed 7.7% with low fear, 38.9% with moderate fear, 48.4% with high fear, and 4.9% with severe fear of childbirth (tokophobia). Of those with tokophobia, 35.7% had primary tokophobia, and 64.3% had secondary tokophobia. Comparative analysis showed no significant differences in most demographic or obstetric characteristics between women with and without severe fear of child birth, although younger age was significantly associated with severe tokophobia**.** Women with severe tokophobia had significantly higher anxiety and stress levels on the DASS-21, while the GAD-7 was not significantly associated after accounting for ordinal trend. The distribution of fear-of-childbirth severity also differed significantly by gravidity, prior vaginal birth, and number of living children. In multivariable analysis, increasing anxiety severity on the DASS-21 remained independently associated with severe tokophobia, whereas stress and GAD-7 categories did not.

**Conclusion:**

This study highlights the significant presence of tokophobia among pregnant women in Lagos, aligning with global evidence while emphasizing the need for culturally tailored research. Routine screening for tokophobia using standardized tools and assessment of anxiety symptoms during antenatal care should be considered for integration into antenatal care, along with targeted counseling and psychological support services following further research.

## Introduction

Pregnancy is a major physical, psychological, and social event in a woman’s life [1]. Ideally, this privileged responsibility of bearing another human life should be a joyous experience. However, for some women, pregnancy becomes a source of significant worry and fear, potentially escalating to a pathological condition known as tokophobia [1]. Tokophobia, first described in the literature by Knauer in 1897, represents an intense fear of childbirth [2]. Primary tokophobia occurs in women who have never experienced birth and may begin during adolescence or after becoming pregnant [3,4]. It can also be seen in women who have been sexually assaulted or raped. Secondary tokophobia occurs in women who have previously experienced pregnancy and birth, often resulting from traumatic labor and birth [4,5]. However, it can also affect women who had normal births, miscarriages, stillbirths, pregnancy terminations, or failed fertility treatments [4].

The prevalence of tokophobia varies significantly in Western countries, with studies reporting rates between 2% and 15% [6]. These variations are likely due to differences in the operationalization of tokophobia, methodological issues, and the scales used in various studies [3]. Severe and debilitating symptoms of childbirth-related fears affect about 20% to 25% of women [7,8]. Symptoms of tokophobia include sleep disturbances, panic attacks, nightmares, avoidance behaviors, anxiety, depression, and extreme fear of birth defects, stillbirth, or maternal death [1,3]. Psychosocial problems and mental ill-health are considerable among women who fear childbirth [9]. Moreover, women with tokophobia who undergo caesarean delivery may experience higher morbidity than those without tokophobia. Studies from Western countries associate tokophobia with adverse obstetric outcomes and postpartum mental health difficulties [8].

Reliable and valid identification of high levels of fear early in pregnancy could enable interventions to support and manage these concerns, reducing distress and fear of childbirth [8]. Effective support can come from family members, friends, obstetricians, midwives, psychologists, or counselors [10]. Some studies have shown a reduction in caesarean birth rates and fear symptoms when women receive psychological and prenatal support [11,12].

While tokophobia is not limited to North America or Europe and has been reported in other parts of the world, its burden in sub-Saharan Africa remains largely hidden. Research on tokophobia in Africa is sparse, highlighting a significant gap in understanding its prevalence and impact in this region.

Despite growing recognition of the psychological dimensions of pregnancy, tokophobia screening remains poorly integrated into routine antenatal care in Nigeria and other low- and middle-income countries. The focus of maternal health services in these settings has largely centered on reducing physical morbidity and mortality, with limited attention paid to mental health challenges such as childbirth-related fear. Yet, tokophobia can lead to serious consequences—including avoidance of vaginal delivery, increased demand for unnecessary cesarean sections, and long-term psychological distress—all of which carry implications for maternal well-being and healthcare resource use [8]. These effects are especially critical in resource-limited settings where access to surgical delivery and mental health services may be constrained.

Understanding the prevalence and correlates of tokophobia in Nigeria is therefore essential for informing clinical guidelines, shaping policy interventions, and tailoring antenatal care to meet the emotional and psychological needs of pregnant women. By exploring both the psychological factors (such as anxiety and stress) and demographic characteristics associated with severe childbirth fear, this study addresses a significant knowledge gap. It also contributes context-specific evidence needed to develop scalable, culturally appropriate mental health screening and support services for women navigating pregnancy in Lagos and similar urban settings in sub-Saharan Africa.

This study aimed to determine the prevalence of tokophobia and its associated protective, risk, and psychological factors in Lagos, Nigeria.

## Materials and methods

This was a cross-sectional survey conducted over a 4-month period between 1^st^ of September, 2023 and 31^st^ of December, 2023. Eight hundred and fifty-five (855) consenting pregnant women were evenly recruited from the antenatal clinics of the 57 flagship, government-owned, primary health care centers (PHCs) across Lagos. Participants were selected using a purposive quota-based sampling strategy to ensure geographic and demographic representation across all 57 flagship Primary Health Care Centers in Lagos State. These PHCs are distributed across the state’s 20 constitutionally recognized Local Government Areas (LGAs) and 37 Local Council Development Areas (LCDAs), which together form the state’s decentralized administrative and health service delivery units. From each PHC, 15 pregnant women were consecutively recruited during routine antenatal visits, allowing each administrative unit to contribute equally to the overall sample. This 15-women-per-center target was pre-specified in our study protocol. While formal demographic stratification was not employed, this approach ensured broad and inclusive coverage across the state’s diverse population. The study included pregnant women presenting for antenatal care at the study locations who were available and willing to participate in the study. Women unable to consent and those who were being treated for known mental health conditions were excluded.

Participants were recruited on-site at the 57 government-owned flagship PHCs across Lagos State during routine antenatal clinic sessions. At each center, trained research assistants approached eligible pregnant women in the antenatal waiting areas, provided a brief explanation of the study purpose, and invited them to participate. Those who expressed interest were screened for eligibility, and written informed consent was obtained prior to data collection.

From each PHC, 15 eligible and consenting pregnant women were recruited, totaling 855 participants. All interviews were conducted in designated private consultation rooms within the PHCs to ensure participant confidentiality and comfort. No participants declined participation or dropped out once consent was obtained, resulting in a 100% response rate.

Data were collected using a structured, interviewer-administered questionnaire during the antenatal visits. The questionnaire was not formally translated into local languages; however, this was mitigated by the use of trained interviewers who were fluent in local dialects and culturally competent. They were extensively trained to ensure consistent interpretation and accurate delivery of the questionnaire items. This approach allowed them to clarify any questions using simple, locally understood terms, thereby ensuring full comprehension and maintaining data quality.

Each interview session lasted approximately 20–30 minutes, depending on the participant’s response pace. No qualitative data were collected, and no audio recordings or transcriptions were made, as the study was strictly quantitative in design.

The questionnaire included sections on biodata, obstetric and psychological characteristics of the study population. Key components incorporated into the questionnaire were the Wijma Delivery Expectancy Questionnaire (W-DEQ A) to measure fear of childbirth, the Depression Anxiety Stress Scales (DASS-21) to evaluate levels of depression, anxiety, and stress, and the Generalized Anxiety Disorder 7-item (GAD-7) scale to assess general anxiety. This thoroughly validated instrument facilitated a detailed analysis of the factors associated with tokophobia among the participants.

The W-DEQ A is a tool used to assess fear of childbirth. Scores from the W-DEQ in this study were interpreted as follows: The W-DEQ A consists of 33 items, each rated on a 6-point Likert scale ranging from 0 to 5 [13]. The total score can range from 0 to 165, with higher scores indicating greater fear of childbirth. Interpretation of Scores: Low Fear (scores 0–37), Moderate Fear (scores 38–65), High Fear (scores 66–84), and Severe Fear (Tokophobia) (85–165) [13].

Psychological symptoms were assessed using the Depression, Anxiety, and Stress Scale – 21 (DASS-21) and the Generalized Anxiety Disorder 7-item (GAD-7) scale.

The DASS-21 is a 21-item self-report questionnaire designed to measure the emotional states of depression, anxiety, and stress [14]. Each of the three scales contains 7 items, and respondents rate the extent to which they have experienced each symptom over the past week on a 4-point scale ranging from 0 (did not apply to me at all) to 3 (applied to me very much or most of the time). The scores for each scale are summed, with higher scores indicating greater severity of symptoms. Interpretation of scores is as follows: for depression, 0–4 is normal, 5–6 is mild, 7–10 is moderate, 11–13 is severe, and 14 + is extremely severe; for anxiety, 0–3 is normal, 4–5 is mild, 6–7 is moderate, 8–9 is severe, and 10 + is extremely severe; and for stress, 0–7 is normal, 8–9 is mild, 10–12 is moderate, 13–16 is severe, and 17 + is extremely severe.

The GAD-7 scale is a brief self-report questionnaire used to identify and assess the severity of generalized anxiety disorder [15]. It consists of 7 items that measure symptoms such as nervousness, inability to control worrying, and restlessness over the past two weeks. Respondents rate the frequency of these symptoms on a 4-point scale ranging from 0 (not at all) to 3 (nearly every day). The total score is calculated by summing the responses, with higher scores indicating greater severity of anxiety. Interpretation of the scores is as follows: 0–4 indicates minimal anxiety, 5–9 indicates mild anxiety, 10–14 indicates moderate anxiety, and 15–21 indicates severe anxiety.

Data entry, cleaning, and analysis were performed using SPSS version 25 (SPSS, Statistics for Windows, IBM Corp, Armonk, NY, USA). The prevalence of the various degrees of fear-of-childbirth was calculated. Bivariate relationships between sociodemographic variables, clinical variables, and tokophobia were tested using appropriate parametric tests. For variables with an inherent ordinal structure, trend-based tests such as the Mantel-Haenszel linear-by-linear association test were utilized to maintain statistical power. Significant independent relationships were assessed using multivariable logistic regression models, where ordinal predictors were modeled as linear terms to account for the trend across severity categories. Odds ratios and confidence intervals were calculated as appropriate and a p-value less than 0.05 was considered statistically significant.

Although the sample size was largely driven by feasibility and the goal of geographic representation, its adequacy can be considered in terms of the precision of the prevalence estimate. Assuming a conservative prevalence of tokophobia of 50%, a 95% confidence level, and a 5% margin of error, a minimum sample size of 384 participants would be required for a simple random sample. The achieved sample size of 855 exceeds this threshold and therefore allows for more precise estimation of prevalence and more stable assessment of associated factors.

Moreover, recruiting participants across 57 PHCs allowed for a more diverse sample, thereby improving generalizability and enhancing representation of the broader population of pregnant women in Lagos.. While clustering effects were considered in the study design by distributing participants evenly across 57 PHCs, statistical adjustment for intra-cluster variability was not formally implemented. Although our analyses did not apply multilevel or robust standard error techniques, the geographic spread and balanced sampling approach help mitigate the risk of site-level bias. The study was conducted in line with the Helsinki Declaration. Permission was obtained from the Lagos State Primary Health Care Board, Lagos State Ministry of Health (LS/PHCB/DPRS//VOL.I/079) to conduct the study in the state’s 57 flagship PHCs.

Ethical approval was obtained from the Health Research and Ethics Committee Lagos State University Teaching Hospital (Reg. No. NHREC04/04/2008) with study reference number.: LREC/06/10/1749. Written informed consent was obtained from all participants.

Informed consent was obtained from all participants prior to enrollment. Trained research assistants explained the study objectives, procedures, and ethical considerations in clear, culturally appropriate language and addressed any questions. Consent was obtained in private consultation rooms to ensure confidentiality and minimize coercion. Written consent was documented only after participants demonstrated full understanding, with explanations provided in local dialects when necessary. The process adhered to approved ethical protocols and was consistently applied across all recruitment sites.

## Results

Socio-demographic characteristics: In this study, the mean age of the women was 28.37 years (SD ± 4.99), with the largest age group being 25–29 years (33.6%). Most participants had secondary education (51.1%) and were married (91.6%). Regarding monthly income, a significant proportion earned less than 35 USD (63.2%), with no participants earning above 300 USD (Table 1).

**Table 1:** Demographic and socio-economic characteristics of the women.

Variables	Frequency	Percentage
Age (years)		
15-19	15	1.8
20-24	194	22.7
25-29	287	33.6
30-34	260	30.4
35 and above	99	11.6
Mean Age ± SD	28.37 ± 4.99	
Educational Status		
None	3	0.4
Primary	57	6.7
Secondary	437	51.1
Tertiary	358	41.9
Marital Status		
Married	783	91.6
Widowed	4	0.5
Separated	5	0.6
Single	63	7.4
Employment Status		
Employed	182	21.3
Self employed	522	61.1
House wife	66	7.7
Unemployed	85	9.9
Religion		
Christianity	554	64.8
Islam	288	33.7
Others	13	1.5
Average Monthly Income		
Less than 35 USD	533	63.2
35–64 USD	246	29.2
65–300 USD	64	7.6
Above 300 USD	0	0

SD – Standard deviation, USD – United States Dollars.

### Obstetric characteristics

The obstetric profile of the pregnant women in this study, as seen in Table 2, shows that 31.7% were primigravida, while 68.3% were multigravida, with a mean gravidity of 2.27 (SD ± 1.22). Most participants were in their third trimester (58.8%). For previous births attended by a doctor, 31.0% had none, 19.9% had one, 11.5% had two, and 3.4% had three or more. Regarding previous births attended by a midwife, 19.1% had none, 22.1% had one, 17.0% had two, and 7.6% had three or more (Table 2).

**Table 2:** Obstetric profile of the pregnant women.

Variables	Frequency	Percentage
Gravidity		
Primigravida	271	31.7
Multigravida	584	68.3
Mean gravidity ± SD	2.27 ± 1.22	
Trimester during the study		
First trimester	86	10.1
Second trimester	266	31.1
Third trimester	503	58.8
Number of living children		
0	316	37.0
1	222	26.0
2	205	24.0
3 and above	112	13.1
Number of vaginal births		
0	303	35.4
1-2	439	51.3
3 and above	113	13.2
Mean number of vaginal births	1.18 ± 1.11	
Number of Cesarean births		
None	844	98.7
1-2	11	1.3
Number of previous births attended by a doctor		
None	265	31.0
1	170	19.9
2	98	11.5
3 and above	29	3.4
Number of previous births attended by a midwife		
None	163	19.1
1	189	22.1
2	145	17.0
3 and above	65	7.6

### Psychological characteristics

The psychological profile of the pregnant women in this study (Table 3) reveals that 4.1% had experienced prior sexual abuse, and 4.8% had faced prior physical assault by a partner. A history of psychiatric illness was reported by 4.6% of the participants. Among these, 1.4% had been diagnosed with depression, 0.9% with anxiety, and 0.5% each with schizophrenia, personality disorder, panic attacks, and bipolar disorder. Anxiety levels, assessed using the Generalized Anxiety Disorder 7-item scale, showed that 72.6% had minimal or no anxiety, 18.9% had mild anxiety, 6.2% had moderate anxiety, and 2.2% had severe anxiety.

**Table 3:** Psychological profile of the pregnant women.

Prior Sexual Abuse		
No	820	95.9
Yes	35	4.1
Prior Physical Assault by Partner		
No	814	95.2
Yes	41	4.8
History of prior psychiatry illness		
Yes	39	4.6
No	816	95.4
Type of psychiatric disorder previously diagnosed		
Nill	816	95.4
Schizophrenia	4	0.5
Personality disorder	4	0.5
Panic attacks	4	0.5
Depression	12	1.4
Anxiety	8	0.9
Bipolar disorder	4	0.5
Others	3	0.4
General anxiety disorder assessment^*a*^		
Minimal or no anxiety	621	72.6
Mild anxiety	162	18.9
Moderate anxiety	53	6.2
Severe anxiety	19	2.2
DASS-21, Depression subscale		
Normal	773	90.4
Mild	31	3.6
Moderate	39	4.6
Severe	7	0.8
Extremely Severe	5	0.6
DASS-21, Anxiety subscale		
Normal	686	80.2
Mild	78	9.2
Moderate	39	4.6
Severe	26	3.0
Extremely Severe	26	3.0
DASS-21, Stress subscale		
Normal	801	93.7
Mild	30	3.5
Moderate	18	2.1
Severe	6	0.7
Extremely Severe	0	0.0
Fear of childbirth^*b*^		
Low fear of childbirth	66	7.7
Moderate fear of childbirth	333	38.9
High fear of childbirth	414	48.4
Severe fear of childbirth (Tokophobia)	42	4.9
Mean fear of childbirth score ± SD^*b*^	64.00 ± 16.6	
Type of severe tokophobia (n = 42)		
Primary Tokophobia	15	35.7%
Secondary Tokophobia	27	64.3%

*a-*Assessed using the Generalized Anxiety Disorder 7-item scale.

*b-*Assessed using Wijma Delivery Expectancy Questionnaire (W-DEQ A),

DASS-21 = The Depression Anxiety Stress Scales.

**Fig 1:**
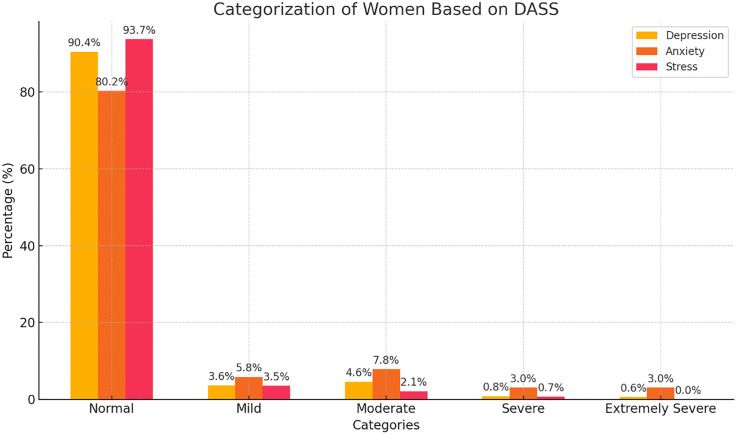
Distribution of the women based on Depression Anxiety Stress Scales (DASS) score.

Depression, assessed using the DASS-21 subscale, was normal in 90.4% of the participants, with 3.6% experiencing mild, 4.6% moderate, 0.8% severe, and 0.6% extremely severe depression (Fig 1). Anxiety subscale scores were normal in 80.2%, mild in 9.2%, moderate in 4.6%, severe in 3.0%, and extremely severe in 3.0% (Fig 1). Stress subscale scores were normal in 93.7%, mild in 3.5%, moderate in 2.1%, and severe in 0.7%, with no cases of extremely severe stress (Fig 1). Tokophobia levels showed 7.7% with low fear, 38.9% with moderate fear, 48.4% with high fear, and 4.9% with severe fear of childbirth, with a mean WDEQ fear of childbirth score of 64.00 (SD ± 16.6). Of those with severe tokophobia, 35.7% had primary tokophobia, and 64.3% had secondary tokophobia.

**Fig 2:**
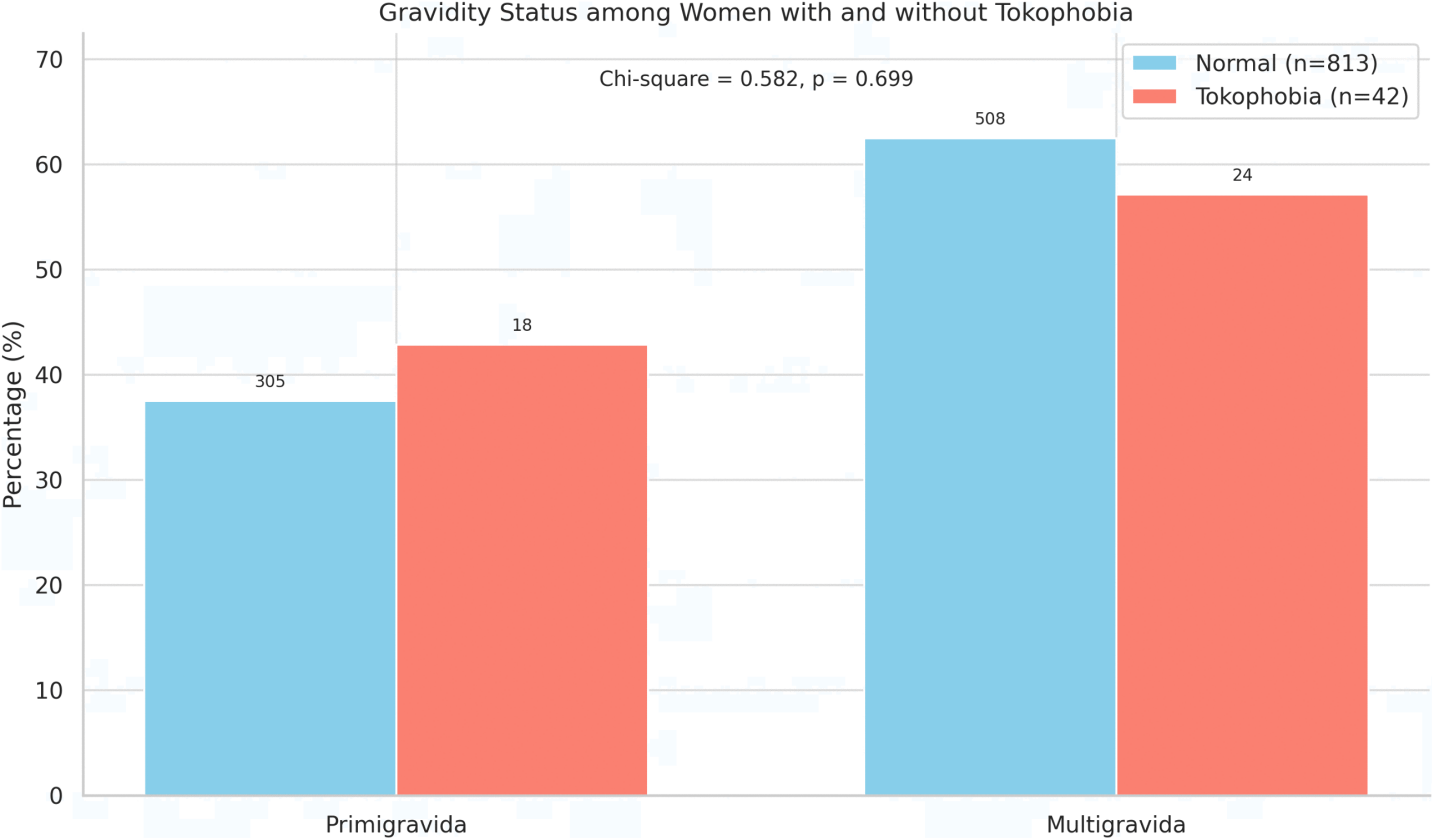
Distribution of gravidity status among women with and without tokophobia.

Bivariate analysis: Among women with tokophobia, 42.9% were primigravida and 57.1% were multigravida, compared to 37.5% and 62.5% respectively among those without tokophobia. Although a slightly higher proportion of tokophobic women were primigravida, this difference was not statistically significant (χ² = 0.582, p = 0.699) (Fig 2).

A comparative analysis of pregnant women with and without severe fear of childbirth (tokophobia) revealed no significant differences in educational status, average monthly income, trimester, prior vaginal birth, cesarean birth, number of living children, prior miscarriage, preterm birth, sexual abuse, physical assault by a partner, or history of psychiatric illness (Table 4). A significant trend was observed for age, with younger women being more likely to have severe tokophobia (p = 0.047). However, significant differences were also found in anxiety levels measured by the DASS-21 Anxiety subscale and stress levels measured by the DASS-21 Stress subscale. Women with severe tokophobia were more likely to have higher levels of anxiety and stress compared to those without severe tokophobia (p < 0.001 for anxiety and p = 0.020 for stress). Specifically, 59.5% of women with severe tokophobia had normal anxiety levels compared to 81.3% of those without severe tokophobia, while 9.5% had extremely severe anxiety compared to 2.7% (Table 4). In contrast, the General Anxiety Disorder 7-item scale did not show a statistically significant difference between the two groups when ordinal trend was accounted for (p = 0.070).

**Table 4:** Comparative analysis of pregnant women with and without Tokophobia.

Variables	Normal n = 813 (%)	Tokophobia^c^ n = 42 (%)	X^2^	p-value
Age (years)			3.946 ^*d*^	0.047
15-19	14 (1.7)	1 (2.4)		
20-24	182 (22.4)	12 (28.6)		
25-29	269 (33.1)	18 (42.9)		
30-34	251 (30.9)	9 (21.4)		
35 and above	97 (11.9)	2 (4.8)		
Educational Status			0.414^*d*^	0.520
None	2 (0.2)	1 (2.4)		
Primary	55 (6.8)	2 (4.8)		
Secondary	419 (51.5)	18 (42.9)		
Tertiary	337 (41.5)	21 (50.0)		
Average Monthly Income			1.776 ^*d*^	0.183
Less than 35 USD	511 (63.7)	22 (53.7)		
35–64 USD	231 (28.8)	15 (36.6)		
65–300 USD	60 (7.5)	4 (9.8)		
Above 300 USD	0 (0.0)	0 (0.0)		
Trimester during the study			2.049 ^*b*^	0.346
First trimester	82 (10.1)	4 (9.5)		
Second trimester	257 (31.6)	9 (21.4)		
Third trimester	474 (58.3)	29 (69.0)		
Prior vaginal birth			0.136	0.742
No prior vaginal birth	287 (35.3)	16 (38.1)		
One or more prior vaginal birth	526 (64.7)	26 (61.9)		
Prior cesarean birth			0.417 ^*b*^	0.427
No prior cesarean birth	803 (98.8)	41 (97.6)		
One or more prior cesarean birth	10 (1.2)	1 (2.4)		
Number of living children			0.024	0.498
No living child	300 (36.9)	16 (38.1)		
One or more living child	513 (63.1)	26 (61.9)		
Prior miscarriage			0.929 ^*b*^	0.574
No prior miscarriage	739 (90.9)	40 (95.2)		
One or more prior miscarriage	74 (9.1)	2 (4.8)		
Prior preterm birth			0.748 ^*b*^	0.366
No prior preterm birth	805 (99.0)	41 (97.6)		
One or more prior preterm birth	8 (1.0)	1 (2.4)		
Prior sexual abuse			0.050 ^*b*^	0.688
No	780 (95.9)	40 (95.2)		
Yes	33 (4.1)	2 (4.8)		
Prior physical assault by partner			0.564 ^*b*^	0.716
No	773 (95.1)	41 (97.6)		
Yes	40 (4.9)	1 (2.4)		
History of psychiatric illness			0.676 ^*b*^	0.433
No	777 (95.6)	39 (92.9)		
Yes	36 (4.4)	3 (7.1)		
DASS-21, Depression subscale			2.228 ^*d*^	0.136
Normal	738 (90.8)	35 (83.4)		
Mild	27 (3.3)	4 (9.5)		
Moderate	36 (4.4)	3 (7.1)		
Severe	7 (0.9)	0 (0.0)		
Extremely Severe	5 (0.6)	0 (0.0)		
DASS-21, Anxiety subscale			13.046 ^*d*^	0.001
Normal	661 (81.3)	25 (59.5)		
Mild	71 (8.7)	7 (16.7)		
Moderate	36 (4.4)	3 (7.1)		
Severe	23 (2.8)	3 (7.1)		
Extremely Severe	22 (2.7)	4 (9.5)		
DASS-21, Stress subscale			5.443 ^*d*^	0.020
Normal	766 (94.2)	35 (83.3)		
Mild	25 (3.1)	5 (11.9%)		
Moderate	16 (2.0)	2 (4.8%)		
Severe	6 (0.7)	0 (0.0)		
Extremely Severe	0 (0.0)	0 (0.0)		
General anxiety disorder assessment^*a*^			3.276^*d*^	0.070
Minimal or No anxiety	599 (73.7)	22 (52.4)		
Mild anxiety	145 (17.8)	17 (40.5)		
Moderate anxiety	51 (6.3)	2 (4.8)		
Severe anxiety	18 (2.2)	1 (2.4)		

*a*-Assessed using the Generalized Anxiety Disorder 7-item scale *b*-Fisher’s exact test applied, X^2^ – chi square, DASS-21 = The Depression Anxiety Stress Scales, *c*- diagnosed with W-DEQ score ≥ 85,

W-DEQ = Wijma Delivery Expectancy Questionnaire, *d*- Linear-by-linear (Mantel–Haenszel) trend test applied for ordinal variables.

Table 5 shows the distribution of fear-of-childbirth severity across selected baseline obstetric variables. The overall distribution differed significantly by gravidity, prior vaginal birth, and number of living children (all p < 0.001), but not by prior cesarean birth (p = 0.505). Primigravida women had a higher proportion of low fear, whereas multigravida women had a higher proportion of high fear. Severe fear of childbirth (tokophobia) was present in both primigravida and multigravida women.

**Table 5:** Distribution of fear-of-childbirth severity by selected baseline obstetric variables.

Variables	Low fear n = 66 (%)	Moderate fear n = 333 (%)	High fear n = 414 (%)	Severe fear n = 42 (%)	Test	p-value
**Gravidity**					12.252^d^	<0.001
Primigravida	39 (12.1)	135 (41.8)	131 (40.6)	18 (5.6)		
Multigravida	27 (5.1)	198 (37.2)	283 (53.2)	24 (4.5)		
**Prior vaginal birth**					16.331^d^	<0.001
No prior vaginal birth	38 (12.5)	130 (42.9)	119 (39.3)	16 (5.3)		
One or more prior vaginal birth	28 (5.1)	203 (36.8)	295 (53.4)	26 (4.7)		
**Prior cesarean birth**					0.444^b^	0.505
No prior cesarean birth	64 (7.6)	329 (39.0)	410 (48.6)	41 (4.9)		
One or more prior cesarean birth	2 (18.2)	4 (36.4)	4 (36.4)	1 (9.1)		
**Number of living children**					17.317^d^	<0.001
No living child	40 (12.7)	134 (42.4)	126 (39.9)	16 (5.1)		
One or more living child	26 (4.8)	199 (36.9)	288 (53.4)	26 (4.8)		

b- Fisher’s exact test applied. d- Linear-by-linear (Mantel–Haenszel) trend test applied for ordinal outcome. Percentages are row percentages. Severe fear of childbirth = tokophobia.

Table 6 presents the multivariable logistic regression analysis, modeling the psychological predictors as ordinal terms to reflect their inherent severity categories. In this model, the DASS-21 Anxiety subscale was found to be significantly and independently associated with the odds of tokophobia (aOR = 1.48, 95% CI: 1.10–2.01, p = 0.011). This indicates that for every one-category increase in anxiety severity (e.g., from Moderate to Severe), the odds of having tokophobia increase by approximately 48%. In contrast, after adjusting for other factors, neither the DASS-21 Stress subscale (p = 0.812) nor the General anxiety disorder assessment (p = 0.592) showed a significant independent association with tokophobia.

**Table 6:** Multivariate analysis evaluating for possible associations with tokophobia.

Predictor Variable	aOR	95% CI	p-value
DASS-21, Anxiety subscale^d^			0.011
Normal	1	ref	
Mild	2.13	0.086–5.28	
Moderate	1.60	0.42–6.07	
Severe	2.63	0.65–10.63	
Extremely Severe	3.35	0.71–15.80.	
DASS-21, Stress subscale^d^			0.812
Normal	1	Ref	
Mild	1.65	0.16–16.55	
Moderate	1.83	0.16–21.31	
Severe	2.12	0.14–31.27	
Extremely Severe	–	–	–
General anxiety disorder assessment^*a,d*^			0.592
Minimal or No anxiety	1	Ref	
Mild anxiety	1.00	0.31–3.18	
Moderate anxiety	1.50	0.45–5.03	
Severe anxiety	2.20	0.53–9.13	

aOR – adjusted odds ratio, CI – confidence interval, *a*-Assessed using the Generalized Anxiety Disorder 7-item scale, d- variables modeled as ordinal terms, p-values represent tests for trend across increasing severity categories.

## Discussion

The socio-demographic characteristics of the study population provide important context for interpreting the findings. In this study, the mean age of the women was 28.37 years (SD ± 4.99), with the largest age group being 25–29 years (33.6%). Most participants had secondary education (51.1%) and were married (91.6%). Regarding monthly income, a significant proportion earned less than 35 USD (63.2%), with no participants earning above 300 USD. These factors may influence perceptions of childbirth and contribute to variations in tokophobia observed in this study. This study investigated the prevalence and associated psychological correlates of severe fear of childbirth (tokophobia) among pregnant women attending antenatal care in Lagos, Nigeria. The main findings indicate that nearly 5% of the women experienced severe fear of childbirth, with the majority of these cases being secondary tokophobia.

Additionally, trend-based bivariate analysis showed that women with severe tokophobia were significantly more likely to report higher levels of anxiety and stress on the DASS-21 scales, while the association with the GAD-7 scale was no longer statistically significant when the ordinal nature of the categories was taken into account. A significant age trend was also observed, with younger women being more likely to have severe tokophobia. In multivariate analysis, increasing DASS-21 anxiety category remained independently associated with severe tokophobia, whereas DASS-21 stress and GAD-7 categories did not. Most other sociodemographic and obstetric factors were not significantly associated with severe tokophobia in the binary comparison.

These findings suggest that psychological factors, particularly anxiety, may be associated with fear of childbirth, but the strength of these associations differs depending on the scale used and whether ordinal severity is taken into account. This aligns with prior research, including studies which reported elevated anxiety symptoms among women with tokophobia but cautioned against assuming causality [6,12]. The high prevalence of moderate to high fear of childbirth in our population (over 87%) also reflects a broader burden of childbirth-related fear, which may be influenced by personal birth experiences, healthcare infrastructure, and cultural narratives around childbirth in Nigeria.

Although most socioeconomic characteristics were not associated with severe tokophobia, the observed age trend and the differences in fear-of-childbirth severity across some obstetric characteristics suggest that vulnerability may not be distributed entirely at random. These insights point to the need for routine psychological screening during antenatal care, with a particular focus on anxiety assessment and management, while also paying attention to women with elevated stress symptoms and those who may be younger or have differing prior birth experiences.

Our community-based survey on tokophobia revealed a prevalence of severe fear of childbirth (tokophobia) of 4.9%, using the Wijma Delivery Expectancy/Experience Questionnaire (W-DEQ) with a cut-off score of ≥85. Nilsson et al., in a systematic review, observed that the most frequently-used scale was the W-DEQ and that country rates (as measured by seven studies using W-DEQ with ≥85 cut-off point) varied from 6.3% to 14.8% [16]. They concluded that rates of severe fear of childbirth, measured in the same way, varied in different countries and recommended the use of the W-DEQ tool with the same cut-off, which was adopted in our study. Our prevalence rate is slightly lower than this range, which may be attributed to cultural differences, varying healthcare practices, or differing levels of support provided to pregnant women in Lagos.

Contrastingly, Nath et al. [17] found a much lower prevalence estimate of tokophobia at 0.032% in London, although they did not use the W-DEQ tool, underscoring the importance of standardized assessment tools for comparability. Demsar et al. reported that 25% of women attending parenting and birth classes in Slovenia exhibited high or very high fear of childbirth, with 1.6% experiencing pathological fear [12]. The lower prevalence in their study compared to ours might be due to differences in study populations, as most of their participants were nulliparous. O’Connell et al.’s meta-analysis found a wide range of tokophobia prevalence rates between 3.7% and 43%, with an overall pooled prevalence of 14% [6]. Our finding of 4.9% fits within this broad range but is closer to the lower end, highlighting the variability due to different definitions and measurement tools.

In the Nigerian context, Esan et al. did not evaluate the prevalence of tokophobia in their qualitative exploratory study [18], but Eleke et al. [19] found only 2.8% of moderate tokophobia among mothers in Port Harcourt, suggesting regional differences within Nigeria. Our higher prevalence rate in Lagos might reflect urban versus regional disparities or differences in healthcare settings, as Eleke’s study was conducted in a single tertiary health facility while ours was in multiple community-based primary health centres.

In terms of demographic characteristics, our study population had a mean age of 28.37 years, predominantly with secondary education and a high rate of self-employment. Our obstetric profile revealed that the majority of women were multigravida and in their third trimester, which aligns with the findings by Ekele et al, who published respondents mean age of 28.1 ± 4.7years old and 55% multiparity rate [19]. Our findings are in line with this Nigerian study [19], where comparable demographic profiles have been reported. The high rate of self-employment could be a reflection of the economic structure in Lagos, where self-owned businesses relatively thrive better when compared to other states.

In our study, 35.7% of the women with tokophobia had primary tokophobia, while 64.3% had secondary tokophobia, indicating that a larger proportion had experienced previous deliveries. Despite this, there was no significant difference in the number of women with tokophobia who had one or more vaginal (p = 0.742) or cesarean births (p = 0.427) compared to those with no prior births. The distribution of fear-of-childbirth severity across obstetric characteristics (Table 5) provides additional clue into the role of parity in tokophobia. As expected, severe fear of childbirth among primigravida women corresponds to primary tokophobia, while that among multigravida women reflects secondary tokophobia. Although multigravida women demonstrated a higher proportion of high fear overall, the proportion of severe fear (tokophobia) was comparable between primigravida and multigravida women. Using an ordinal trend-based analysis, the overall distribution of fear-of-childbirth severity differed significantly by gravidity, prior vaginal birth, and number of living children, but not by prior cesarean birth. This suggests that severe fear of childbirth is not confined to women with prior birth experiences and may arise both in anticipation of a first delivery and following previous pregnancies. These findings support the concept that tokophobia is multifactorial and not solely determined by obstetric history. This finding contrasts with Sioma-Markowska’s research, which demonstrated that successive childbirths significantly impact anxiety levels (p = 0.04217), with the highest anxiety observed in primiparous women [20]. Possible explanations for this discrepancy may include differences in study populations, socio-cultural factors, methods in assessing anxiety and tokophobia or the varying support systems available to women during childbirth.

Comparative analysis showed no significant differences in most demographic or obstetric characteristics between women with and without severe tokophobia, although a significant age trend indicated that younger women were more likely to have severe tokophobia. However, psychological symptoms assessed using the DASS-21 scales showed higher anxiety and stress levels among women with severe tokophobia. This aligns with previous studies that have identified anxiety and stress as significant factors associated with tokophobia [20,21]. This implies that while anxiety and stress may correlate with tokophobia, anxiety appears to have a more robust independent association in our dataset, whereas other factors might also play crucial roles in its development [22]. Potential explanations for these results could include the influence of previous traumatic childbirth experiences, personal coping mechanisms, or varying levels of social and medical support during pregnancy [12,22,23]. Furthermore, although both anxiety and stress levels were higher among women with tokophobia in the bivariate analysis, only anxiety remained statistically significant after adjusting for the other psychological variables in the multivariate model. This pattern may indicate overlap between the measured constructs, residual confounding, cultural stigma affecting self-reporting of psychological symptoms, or limited statistical power given the relatively small tokophobic subgroup**.** These findings highlight the complexity of tokophobia and suggest that other sociodemographic or psychosocial factors not captured in the current model may play an influential role. Given the cross-sectional design of this study, the direction of the relationship between psychological factors such as stress or anxiety and fear of childbirth cannot be established, and reverse causation remains possible.

Therefore, this study has demonstrated the significant presence of tokophobia in Lagos, aligning with some international findings and differing from others, thus emphasizing the importance of culturally tailored research and interventions. Future studies should continue using standardized tools like the W-DEQ and incorporate qualitative insights to provide a comprehensive understanding of tokophobia’s impact on maternal health and decision-making. Practical recommendations include incorporating routine screening for tokophobia into antenatal care using validated tools, along with targeted counseling and psychological support services for high-risk patients. These interventions could reduce anxiety and improve maternal outcomes. Efforts should also focus on training healthcare providers to recognize and address tokophobia, making the findings applicable in similar settings.

Evidence from the WHO underscores the importance of integrating mental health services into routine antenatal care [24]. ACOG also recommends that pregnant patients experiencing significant fear or distress, such as tokophobia, be identified early through validated screening tools and referred for appropriate interventions [25]. Aligning with these guidelines, this study supports the need for proactive identification of tokophobia and the development of scalable intervention programs, particularly in resource-limited settings.

While our design ensured broad geographic and demographic coverage, the non-random selection of participants may limit the extent to which our findings can be extrapolated to all pregnant women in Lagos or other settings. These limitations notwithstanding, the study offers valuable and timely insights into tokophobia in a large urban African setting — an area that has been vastly under-researched — and can inform both future research and public health interventions.

In conclusion, this study identified a 4.9% prevalence of tokophobia among pregnant women attending antenatal care in Lagos, with most cases being secondary tokophobia. Our findings reveal the multifactorial nature of tokophobia and highlight the importance of integrating routine psychological screening into antenatal care, even in the absence of clear risk profiles. Future research should adopt mixed-methods approaches and standardized tools like the W-DEQ to further explore cultural, experiential, and systemic contributors to tokophobia and evaluate targeted interventions in resource-constrained settings.
